# The impact of physical exercise participation on residents’ sense of social justice: an empirical analysis based on the 2023 data of China’s CGSS

**DOI:** 10.3389/fpubh.2026.1932575

**Published:** 2026-10-07

**Authors:** Yanhong Wu, Yihua Zhang, Zihui Liu, Zhirong Zheng, Longan Cao

**Affiliations:** 1 Faculty of International Tourism and Management, City University of Macau, Macao, Macao SAR, China; 2 College of Arts and Sciences, Washington University, St. Louis, MO, United States; 3 School of Physical Education, Southwest University, Chongqing, China; 4 General Education College, Chongqing Vocational College of Applied Technology, Chongqing, China

**Keywords:** CGSS 2023, physical exercise participation, physical health status, sense of social justice, social trust

## Abstract

**Background:**

Elevating residents’ sense of social justice is vital for social stability and harmonious societal development. Existing literature has documented correlations between physical exercise and subjective well-being, social trust and life satisfaction, yet few studies have explored how physical exercise participation aligns with sense of social justice among Chinese residents or unpacked correlational linkages across relevant variables. This cross-sectional analysis addresses this research gap usingdata from a nationwide social survey.

**Methods:**

Data were sourced from the 2023 Chinese General Social Survey (CGSS 2023), with 5,364 respondents retained after data screening and exclusion of missing, abnormal, and out-of-range observations. Multiple linear regression and structural equation modeling (SEM) with Bootstrap testing were applied to analyze synchronous correlations between physical exercise participation and residents’ sense of social justice, as well as correlated mediating chains involving self-rated physical health and social trust. Demographic, socioeconomic, health and social capital indicators were included as control covariates.

**Results:**

The average score of residents’ sense of social justice reached 3.30 (above the medium threshold), and 52.49% of respondents held positive evaluations of societal fairness; the sample-wide physical exercise participation rate stood at 57.11%. After accounting for all control variables, physical exercise participation displayed significant positive correlations with sense of social justice, self-rated physical health and social trust. Both self-rated physical health and social trust were positively correlated with sense of social justice. Bootstrap tests confirmed two independent correlated mediating linkages and one serial correlated mediating chain connecting exercise participation and sense of social justice via physical health and social trust.

**Conclusion:**

Physical exercise participation was positively associated with residents’ sense of social justice. The findings further suggest that physical health status and social trust may help explain the observed association between exercise participation and sense of social justice, highlighting the potential interplay among exercise, health, social relationships, and fairness perceptions. These results underscore the value of considering physical well-being and social trust alongside public fitness participation when seeking to understand residents’ perceptions of sense of social justice. Given the cross-sectional nature of the data, however, the observed relationships should be interpreted as associations rather than causal effects.

## 1 Introduction

The report to the 20th National Congress of the Communist Party of China emphasized that national security underpins national rejuvenation and that social stability is essential to national prosperity. Since residents’ sense of social justice is closely related to social cohesion and stability, identifying the factors associated with fairness perceptions is important for improving social governance and promoting harmonious development.

Physical exercise, as a planned form of physical activity (1), is associated not only with physical fitness and health (2), but also with social connection, communication, and integration (3). Existing studies have primarily examined its associations with individual-level outcomes, including happiness (4, 5), social trust (6, 7), and life satisfaction (8, 9). However, these outcomes do not fully capture individuals’ broader evaluations of the social environment. Happiness and life satisfaction mainly reflect subjective well-being, whereas sense of social justice concerns individuals’ judgments of resource distribution, equality of opportunity, and institutional arrangements.

Sense of social justice reflects individuals’ perceptions and value judgments regarding fairness in the broader social environment (10). It has been conceptualized in terms of outcome and procedural fairness (11), as well as distributive, procedural, and opportunity fairness (12). Although Chinese residents generally report relatively high levels of perceived social fairness (13), substantial differences remain across social groups. Individuals with lower socioeconomic status are more likely to regard income inequality as unfair, and changes in social position are often accompanied by changes in fairness perceptions (14).

Existing research on sense of social justice has largely emphasized structural and socioeconomic determinants. Positive outcomes, comparative payoffs, and transparent procedures have been identified as important correlates of fairness perceptions (15), while judgments of resource distribution, participation opportunities, and evaluation procedures are also closely related to perceived fairness (16). Income inequality is negatively associated with sense of social justice, particularly among low-income and unemployed groups (17), and Internet engagement has also been linked to individuals’ fairness assessments. Although previous studies have reported associations between physical exercise and social fairness, social inclusion, and broader social development (18), differences in cultural context, social institutions, and sporting environments limit the extent to which these findings can be directly generalized to Chinese residents.

More importantly, existing evidence does not fully explain whether and how an everyday behavioral and social-participation factor such as physical exercise is associated with residents’ evaluations of social justice. Previous studies have shown that sense of social justice is embedded in social relationships and is related to motivational, emotional, and cognitive tendencies in interpersonal interaction (19). Physical exercise is associated with greater happiness (20, 21), while CGSS-based research has found positive associations between exercise participation and interpersonal or generalized trust. Exercise combined with social interaction may also be related to more positive emotional experiences and greater vitality (22). However, these studies largely examine health, well-being, trust, and fairness separately and therefore cannot determine whether exercise participation is linked to broader fairness perceptions through an integrated set of health-related and relational resources.

The theoretical contribution of the present study lies not in re-establishing the well-documented associations of physical exercise with health or social trust, but in integrating these relationships into a broader framework of social evaluation. Specifically, this study extends the conventional view of physical exercise as primarily a health-promoting behavior by considering it simultaneously as a behavior embedded in health capital and social capital. From the perspective of Social Capital Theory, physical exercise can be understood not only as an individual health behavior but also as a form of social participation. Exercise participation may provide opportunities for interpersonal contact, cooperation, reciprocity, and the development of social networks. These social experiences may strengthen social connectedness and be associated with more positive evaluations of interpersonal relations and broader social arrangements. Because sense of social justice reflects individuals’ subjective evaluations of resource distribution, opportunities, and social arrangements, greater participation in physical exercise may therefore be positively associated with sense of social justice. Therefore, this study proposes hypothesis 1: Physical exercise participation is positively associated with residents’ sense of social justice.

Health Capital Theory provides a theoretical basis for considering physical health as an intermediate factor linking physical exercise participation with sense of social justice. From this perspective, health represents a form of individual capital that can be accumulated and maintained through health-related behaviors, including physical exercise. Individuals with better health are generally more capable of participating in daily activities and social life and may experience fewer health-related constraints and burdens. Such differences in physical well-being may be associated with how individuals evaluate their living conditions and broader social environment. Consequently, physical exercise participation may be associated with better self-rated physical health, which in turn may correspond to more positive perceptions of sense of social justice.

Studies have shown that participation in physical exercise can reduce the risk of cardiovascular disease, improve blood glucose levels, help control and lower the risk of hypertension, and enhance physical functioning and quality of life (23). Appropriate physical exercise can also significantly reduce mortality risk (24). Accordingly, this study proposes the following hypothesis 2: Self-rated physical health forms an independent correlated mediating linkage between physical exercise participation and sense of social justice.

Social Capital Theory also provides a theoretical explanation for the role of social trust. Social capital is commonly reflected in social networks, norms of reciprocity, and interpersonal trust (25). Participation in physical exercise, especially in socially organized or community-based settings, may provide opportunities for repeated interaction, cooperation, and reciprocal exchange (26). These social experiences may be associated with stronger generalized trust toward others. Social trust, in turn, is closely related to individuals’ evaluations of the social environment because people who perceive others and social institutions as more trustworthy may also evaluate social arrangements as more predictable, legitimate, and fair (27, 28). Accordingly, social trust may represent an important relational mechanism connecting physical exercise participation with sense of social justice. Accordingly, this study proposes the following hypothesis 3: Social trust forms an independent correlated mediating linkage between physical exercise participation and sense of social justice.

Relevant theoretical and correlational evidence further clarifies the sequential linkage among physical health status, social trust and sense of social justice, laying the groundwork for Hypothesis 4 (29). First, Health Capital Theory regards self-rated physical health as fundamental individual endowment, which serves as a prerequisite condition for residents’ sustained social interaction. Individuals with better physical conditions tend to take part in group sports and daily social exchanges more frequently, which correlates with higher generalized social trust; by contrast, poor physical status restricts social engagement and synchronously accompanies lower trust levels. Second, the social cognition logic of social justice perception indicates that physical well-being shapes people’s baseline emotional and cognitive bias, which further correlates with their judgments on the credibility of other people and social institutions. That is, synchronous positive correlations exist from physical health to social trust, and from social trust to sense of social justice. Existing correlational studies also record that social trust presents consistent positive synchronous correlations with residents’ sense of social justice, while physical health status is synchronously associated with the formation of social trust (6, 13). Synthesizing the above theoretical framework and empirical correlational patterns, self-rated physical health and social trust may form a serial correlated mediating chain linking physical exercise participation and sense of social justice. Accordingly, this study proposes Hypothesis 4: Self-rated physical health and social trust form a serial correlated mediating chain in the correlational linkage between physical exercise participation and residents’ sense of social justice, following the correlational sequence: physical exercise participation → self-rated physical health → social trust → sense of social justice.

## 2 Methods

### 2.1 Data source

The data used in this study were obtained from the 2023 Chinese General Social Survey (CGSS 2023). The CGSS is one of the most representative nationwide social survey projects in China. The present analyses were conducted using unweighted data. Although the CGSS adopts a stratified multistage probability sampling design, sampling weights were not applied in the main regression and structural equation models. Therefore, the estimates reported in this study should be interpreted as associations within the analytic sample rather than as strictly population-representative estimates.

In accordance with the research objectives, this study selected variables related to sense of social justice, physical exercise participation, physical health status, social trust, and relevant control variables for analysis. The original CGSS 2023 dataset contained 11,327 observations. After excluding 3,240 cases with missing values in key variables, 413 cases with abnormal or internally inconsistent responses, and 2,310 cases with values outside the predefined valid ranges, a final analytic sample of 5,364 respondents was retained (Figure 1).

**Figure 1:**
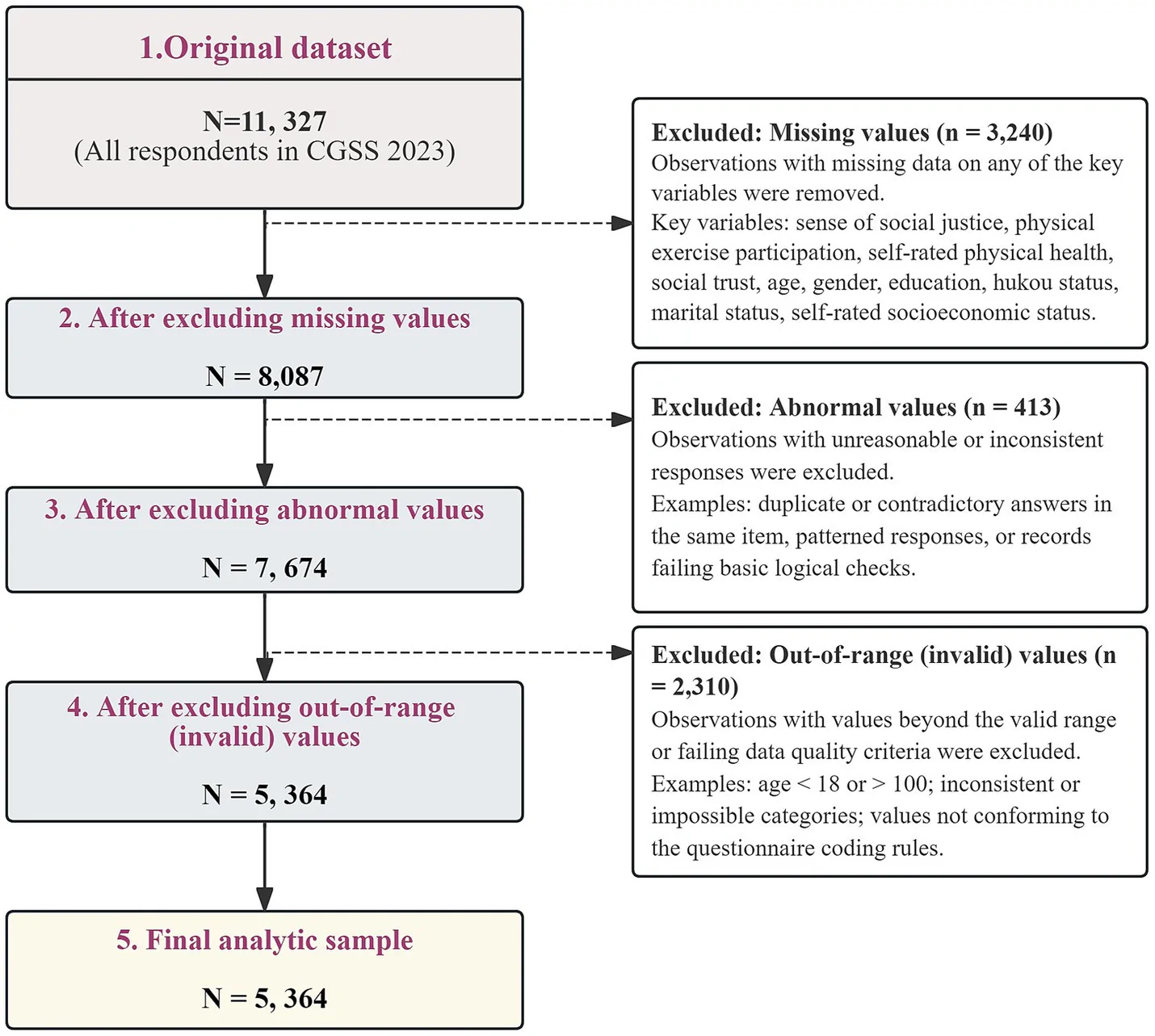
Flowchart of sample screening.

### 2.2 Variable measurement

#### 2.2.1 Sense of social justice

Sense of social justice was measured using item A35 of the CGSS 2023 questionnaire: “Overall, do you think today’s society is fair or unfair?” Responses ranged from 1 (“completely unfair”) to 5 (“completely fair”), with higher scores indicating a stronger perception of sense of social justice. Conceptually, sense of social justice reflects individuals’ subjective evaluations of the fairness of the broader social environment, including their overall judgments of social arrangements, opportunities, and resource distribution. Similar global fairness items have been widely used in Chinese survey research to capture respondents’ overall evaluations of societal fairness. Because this item is intended to measure a global judgment rather than a latent multidimensional justice scale, its use is consistent with the conceptual aim of the present study (30). However, psychometric evidence for this specific single item is more limited than that available for multi-item justice measures.

#### 2.2.2 Physical exercise participation

Physical exercise participation was measured using item A30-9 of the CGSS 2023 questionnaire, which asks respondents how often they participated in physical exercise during their leisure time in the past year. For the main analysis, the measure was operationalized as participation versus non-participation: respondents reporting exercise participation at any frequency were coded as 1, whereas those reporting no participation were coded as 0. This operationalization was adopted because the primary theoretical interest of the study concerns whether engagement in physical exercise is associated with sense of social justice, rather than estimating a dose–response relationship based on exercise frequency. Importantly, the CGSS item captures self-reported frequency of leisure-time exercise but does not provide information on exercise duration, intensity, type, or organizational context. It should therefore be interpreted as an indicator of exercise participation rather than as a measure of compliance with recommended physical activity guidelines. Although this binary coding facilitates a clear distinction between participation and non-participation, it reduces the information contained in the original ordinal frequency categories and may obscure potential dose–response associations between exercise frequency and sense of social justice. Accordingly, the findings should be interpreted as reflecting differences between participants and non-participants rather than differences across levels of exercise frequency. Future studies should retain the original frequency categories or use more detailed measures of exercise frequency, duration, and intensity to examine possible dose–response relationships.

#### 2.2.3 Physical health status

Self-rated physical health was measured using item A15, which asks respondents to evaluate their current health status on a five-point scale, with higher scores indicating better perceived health. Self-rated health provides an integrative subjective assessment of an individual’s overall health condition and is widely used in large-scale population surveys. Evidence from a nationally representative Chinese sample has demonstrated moderate-to-substantial test–retest reliability and predictive validity of single-item self-rated health measures, including their ability to predict subsequent mortality (31). In the present study, it is treated as an indicator of perceived physical health rather than as an objective clinical measure.

#### 2.2.4 Social trust

Social trust was measured using item A33: “In general, do you agree that most people in this society can be trusted?” Responses were recorded on a five-point scale, with higher scores indicating greater trust. Conceptually, this item captures generalized social trust—that is, individuals’ general expectations regarding the trustworthiness of people in society rather than trust toward specific family members, acquaintances, or institutions. The “most people can be trusted” item has a long-established tradition as a global indicator of generalized trust in large-scale national and cross-national surveys, although methodological research also acknowledges limitations related to measurement error and measurement equivalence (32).

#### 2.2.5 Covariates

To reduce potential omitted variable bias, a range of covariates was included in the analyses, including gender, age, ethnicity, educational attainment, household registration (hukou) status, marital status, self-rated socioeconomic status, social security status, and social interaction capacity.

Age was calculated by subtracting the respondent’s birth year from 2023 and was restricted to the range of 18–100 years. Educational attainment was measured according to the respondent’s highest level of education completed. Gender was coded as 1 for male and 2 for female. The measurement and descriptive statistics of the main variables are presented in Table 1.

**Table 1:** Variable definition and descriptive statistics.

Variable category	Variable	Values/meaning	Source item	Mean/proportion
Dependent variable	Sense of social justice	Very unfair = 1; Relatively unfair = 2; Neither fair nor unfair = 3; Relatively fair = 4; Completely fair = 5	A35. Overall, do you think today’s society is fair or unfair?	3.30
Independent variable	Physical exercise participation	Participates in exercise (several times a year or more) = 1; Never participates in exercise = 0	A30-9. In the past year, have you often engaged in the following activities during your leisure time?	57.11%
Demographic characteristics	Gender	Male = 1; Female = 2	A2. Gender	44.74%
	Age	Actual age of the respondent	A3. Your birth year	54.55
	Ethnicity	Ethnic minority = 1; Han = 0	A4. Your ethnicity	9.34%
	Educational attainment	Primary school or below = 1; Junior high school = 2; Senior high school/vocational secondary school = 3; Junior college or above = 4	A7a. What is your highest level of education at present (including current study)?	1 = 34.47% 2 = 29.31% 3 = 18.61% 4 = 17.61%
	Self-rated economic status	Lower = 1; Lower-middle = 2; Middle = 3; Upper-middle = 4; Upper = 5	A43e. Your current socioeconomic status/self-rated economic level	3.71
	Household registration type	Non-agricultural hukou = 1; Agricultural hukou = 0	A18. What is your currenthousehold registration status?	27.66%
	Marital status	Married (first marriage with spouse,separated but not divorced, remarried with spouse) = 1; Single (divorced, widowed, cohabiting, unmarried) = 0	A69. What is your current marital status?	72.14%
Physical health	BMI	Normal BMI (18.5–23.9) = 1; Abnormal BMI = 0	—	52.21%
	Self-rated health status	Very unhealthy = 1; Relatively unhealthy = 2; Average = 3; Relatively healthy = 4; Very healthy = 5	A15. How would you rate your current health status?	3.35
Social capital	Social security status	Participates in at least one social security program = 1; Does not participate in any social security program = 0	A61. Which of the following social security programs do you currently participate in?	96.51%
	Social interaction ability/frequency	Never = 1; Rarely = 2; Sometimes = 3; Often = 4; Very frequently = 5	A31-1. In the past year, have you often done the following in your leisure time—socializing/visiting others?	2.64
	Social trust	Strongly disagree = 1; relatively disagree = 2; neither agree nor disagree = 3; relatively agree = 4; strongly agree = 5	A33. Overall, to what extent do you agree that most people in this society can be trusted?	3.48

The percentages of the dichotomous variables in the table represent the proportion of the sample that chose the option with a value of 1.

### 2.3 Statistical analysis

#### 2.3.1 Multiple linear regression model

Multiple linear regression analysis was conducted using SPSS 25.0 to examine the association between physical exercise participation and sense of social justice among Chinese residents (Hypothesis 1). The model was specified as follows:

$$\text{Sense}=\beta_{0}+\beta_{1\;\text{exercise}\;i}+\beta_{2\;\text{controls}\;i}+\varepsilon_{i}$$ (1)

Where sense denotes the dependent variable, sense of social justice; exercise denotes the core explanatory variable, physical exercise participation; controls represents the set of control variables; *ε* is the random error term; and i denotes the *i*-th sample observation.

#### 2.3.2 Structural equation modeling

Structural equation modeling was performed using AMOS 26.0 to examine the indirect associations linking physical exercise participation with residents’ sense of social justice, specifically through self-rated physical health (Hypothesis 2) and social trust (Hypothesis 3). The model was specified as follows:

y=By+Γx+ζ (2)

Where *y* denotes the vector of endogenous variables, including self-rated physical health, social trust, and perceived social justice; *x* denotes the vector of exogenous variables, including physical exercise participation and the control variables; B represents the coefficient matrix among the endogenous variables; Γ represents the coefficient matrix from the exogenous variables to the endogenous variables; and *ζ* denotes the vector of structural residuals.

Furthermore, all indirect effects were tested using 5,000 Bootstrap resamples in AMOS 26.0. Bias-corrected 95% Bootstrap confidence intervals were calculated for the specific indirect effects through self-rated physical health and social trust and for the serial indirect effect through self-rated physical health and social trust (Hypothesis 4). An indirect effect was considered statistically significant when the confidence interval did not include zero.

## 3 Results and analysis

### 3.1 Sense of social justice and physical exercise participation across different groups

Overall, the proportion of residents who perceived society as fair (52.49%) was substantially higher than the proportion of those who perceived society as unfair (22.83%), with a mean sense of social justice score of 3.30 (Table 1). Regarding the key independent variable, physical exercise participation, 42.89% of residents reported never participating in physical exercise. This proportion was higher than that reported in the CGSS 2021 data (34.88%).

Statistical analyses were conducted to examine sense of social justice and physical exercise participation across different characteristic groups. For self-rated economic status, the category “lower-middle class” was merged into “lower class,” while “upper-middle class” was merged into “upper class”.

The results showed certain differences in sense of social justice across different groups. Specifically, male residents reported slightly higher sense of social justice than female residents; residents aged 70 years and above reported higher levels than those in other age groups; ethnic minority residents reported slightly higher levels than Han residents; residents with primary school education or below reported higher levels than those with other educational backgrounds; residents who rated their economic status as upper class reported higher levels than those who rated themselves as middle or lower class; residents with non-agricultural hukou reported slightly higher levels than those with agricultural hukou; and single residents reported slightly higher sense of social justice than married residents.

In terms of physical exercise participation, men participated more actively than women; residents aged 18–44 participated in physical exercise at a substantially higher rate than those in other age groups; Han residents participated slightly more than ethnic minority residents; residents with a college degree or above were more likely to participate in physical exercise; residents who rated their economic status as upper class showed stronger participation initiative; and residents with non-agricultural hukou and single residents were more active in physical exercise participation (Table 2).

**Table 2:** Sense of social justice and physical exercise participation of residents with different characteristics.

Variable	Group	*n*	Sense of social justice	Participation in physical exercise (%)
Gender	Female	2,964	3.30**	55.83%**
	Male	2,400	3.32**	58.66%**
Age	18–44	1,433	3.22***	73.55%***
	45–69	2,864	3.31***	52.79%***
	70 and above	1,067	3.42***	46.57%***
Ethnicity	Han	4,863	3.29	58.27%**
	Ethnic minority	501	3.43	45.70%**
Educational attainment	Primary school and below	1849	3.42***	34.55%***
	Junior high school	1,572	3.21***	55.47%***
	Senior high school/vocational secondary school	998	3.26***	72.44%***
	Junior college and above	945	3.29***	87.72%***
Self-rated economic status	Upper	295	3.55***	66.10%***
	Middle	2,103	3.45***	61.72%***
	Lower	2,966	3.18***	52.93%***
Household registration type	Agricultural hukou	3,880	3.30*	50.67%***
	Non-agricultural hukou	1,484	3.32*	73.92%***
Marital status	Married	3,870	3.30**	54.65%***
	Single	1,494	3.31**	63.45%***

Differences in mean sense of social justice were assessed using independent-samples t tests or one-way ANOVA, as appropriate; differences in exercise participation proportions were assessed using chi-square tests.

### 3.2 Association between physical exercise participation and residents’ sense of social justice

Using sense of social justice as the dependent variable and physical exercise participation as the core independent variable, regression analyses were conducted after incorporating socioeconomic and demographic characteristics, physical health status, and social capital variables. Regression diagnostics indicated no serious violations of the assumptions underlying the multiple linear regression analyses. Multicollinearity was not a concern, with VIF values ranging from 1.239 to 1.276 and tolerance values ranging from 0.767 to 0.817. Inspection of standardized residual plots revealed no obvious violations of linearity or homoscedasticity, and the residual distribution showed no substantial departure from normality. In addition, the maximum Cook’s distance was 0.37, well below the conventional threshold of 1, suggesting that no single observation exerted undue influence on the regression estimates. The baseline model showed that, after controlling for gender, age, ethnicity, educational attainment, hukou status, marital status, and self-rated economic status, the regression coefficient of physical exercise participation on sense of social justice was 0.055, with a standardized coefficient of 0.026 and a *t*-value of 3.752***, reaching the conventional level of statistical significance (Table 3).

**Table 3:** Results of regression analyses.

Outcome variable	Predictor	*R* ^2^	Adjusted *R*^2^	*F*	B	95% CI	*β*	*t*
Sense of social justice	Participation in physical exercise	0.025	0.023	17.131***	0.055	[0.026, 0.084]	0.026	3.752***
	Gender				−0.029	[−0.085, 0.027]	−0.014	−1.019
	Age				0.063	[0.017, 0.109]	0.041	2.680**
	Ethnicity				0.142	[0.048, 0.236]	0.040	2.949**
	Educational attainment				−0.045	[−0.076, −0.014]	−0.048	−2.851**
	Household registration type				0.011	[−0.055, 0.077]	0.005	0.325
	Marital status				−0.037	[−0.099, 0.025]	−0.016	−1.166
	Self-rated socioeconomic status				0.232	[0.186, 0.278]	0.135	9.845***
Health Status	Participation in physical exercise	0.161	0.160	128.804***	0.300	[0.238, 0.362]	0.131	9.478***
	Gender				−0.074	[−0.131, −0.017]	−0.032	−2.557*
	Age				−0.416	[−0.463, −0.369]	−0.249	−17.428***
	Ethnicity				−0.088	[−0.184, 0.008]	−0.023	−1.799
	Educational attainment				0.113	[0.081, 0.145]	0.109	7.003***
	Household registration type				0.023	[−0.042, 0.088]	0.009	0.691
	Marital status				0.004	[−0.054, 0.062]	0.002	0.135
	Self-rated socioeconomic status				0.250	[0.203, 0.297]	0.132	10.404***
Sense of social trust	Participation in physical exercise	0.022	0.021	15.168***	0.027	[0.016, 0.038]	0.013	4.872***
	Gender				−0.034	[−0.089, 0.021]	−0.016	−1.208
	Age				0.206	[0.160, 0.252]	0.134	8.701***
	Ethnicity				0.146	[0.051, 0.241]	0.041	3.008**
	Educational attainment				0.037	[0.006, 0.068]	0.039	2.336*
	Household registration type				−0.053	[−0.119, 0.013]	−0.023	−1.586
	Marital status				0.082	[0.020, 0.144]	0.035	2.584**
	Self-rated socioeconomic status				0.102	[0.056, 0.148]	0.059	4.316***
Sense of social justice	Participation in physical exercise	0.140	0.138	87.115***	0.023	[0.010, 0.036]	0.011	3.394***
	Gender				−0.012	[−0.065, 0.041]	−0.006	−0.447
	Age				0.027	[−0.018, 0.072]	0.018	1.169
	Ethnicity				0.101	[0.012, 0.190]	0.028	2.222*
	Educational attainment				−0.066	[−0.095, −0.037]	−0.070	−4.414***
	Household registration type				0.027	[−0.035, 0.089]	0.011	0.849
	Marital status				−0.064	[−0.122, −0.006]	−0.028	−2.164*
	Self-rated socioeconomic status				0.179	[0.135, 0.223]	0.104	8.009***
	health level				0.076	[0.051, 0.101]	0.083	6.009***
	Sense of social trust				0.330	[0.305, 0.355]	0.331	25.832***

*p < 0.05, **p < 0.01, ***p < 0.001.

After further including physical health status and social trust, the direct coefficient of physical exercise participation on sense of social justice was 0.023, with a standardized coefficient of 0.011 and a *t*-value of 3.394***, and the result remained statistically significant. These findings indicate that physical exercise participation remained significantly and positively associated with residents’ sense of social justice after adjustment for the included covariates, thereby supporting Hypothesis 1.

The results for the control variables showed that self-rated economic status had a stable positive effect on sense of social justice, indicating that residents with higher socioeconomic status were more likely to perceive society as fairer. In the final model, ethnicity was positively significant, and marital status and educational attainment were also positively significant. Physical health status and social trust were both significantly and positively associated with sense of social justice. In particular, the standardized coefficient of social trust was 0.331. These results suggest that residents’ judgments of sense of social justice are related not only to their subjective and objective socioeconomic status, but also closely to their health status and level of social trust (33).

### 3.3 Mediation analysis

Structural equation modeling (SEM) was conducted using AMOS 26.0 to examine the hypothesized associations among physical exercise participation, self-rated physical health, social trust, and sense of social justice. The results indicated that the model demonstrated a good fit to the data, with *χ*^2^/df = 1.198, CFI = 0.978, TLI = 0.967, NFI = 0.992, RMSEA = 0.015, and SRMR = 0.046. These indices suggest that the specified structural model provided an acceptable representation of the observed data.

This study further examined the indirect associations linking physical exercise participation with sense of social justice through self-rated physical health and social trust. As shown in Table 4, the results showed that physical exercise participation significantly and positively predicted self-rated health status (B = 0.300, *β* = 0.131, *t* = 9.478***), indicating that residents who participated in physical exercise tended to report better health status.

**Table 4:** Bootstrap analysis of mediating effect.

Category	Effect value	Bootstrap SE	95% CI	Relative mediating effect/%
Total indirect effect between participation in physical exercise and sense of social justice	0.032	0.030	[0.003, 0.061]	58.19%
Participation in physical exercise → Self-rated health status → Sense of social justice	0.023	0.005	[0.014, 0.033]	41.81%
Participation in physical exercise → Social trust → Sense of social justice	0.005	0.002	[0.001, 0.010]	9.09%
Participation in physical exercise → Self-rated physical health status → Social trust → Sense of social justice	0.004	0.002	[0.001, 0.007]	7.27%
Direct effect between participation in physical exercise and sense of social justice	0.023	0.029	[0.005, 0.041]	41.81%

Self-rated health status also significantly and positively predicted sense of social justice (B = 0.076, *β* = 0.083, *t* = 6.009***), suggesting a strong positive association between health status and residents’ sense of social justice. The Bootstrap test showed that the indirect effect of “physical exercise participation → self-rated health status → sense of social justice” was 0.023, with a 95% confidence interval of [0.014, 0.033], which did not include zero. This indicates that the mediating effect of physical health status was significant, thereby supporting Hypothesis 2.

Physical exercise participation was significantly and positively associated with social trust (B = 0.027, *β* = 0.013, *t* = 4.872***). In addition, the indirect effect of “physical exercise participation → social trust → sense of social justice” was 0.005, with a 95% confidence interval of [0.001, 0.010]. This indicates that the independent mediating effect of social trust was significant, thereby supporting Hypothesis 3.

Regarding the chain-mediating pathway, the indirect effect of “physical exercise participation → self-rated health status → social trust → sense of social justice” was 0.004, with a 95% confidence interval of [0.001, 0.007], which did not include zero. This finding indicates that physical exercise participation was positively associated with residents’ health status, social trust, and sense of social justice, thereby supporting Hypothesis 4. Based on the structural equation model, thus resulting in Figure 2.

**Figure 2:**
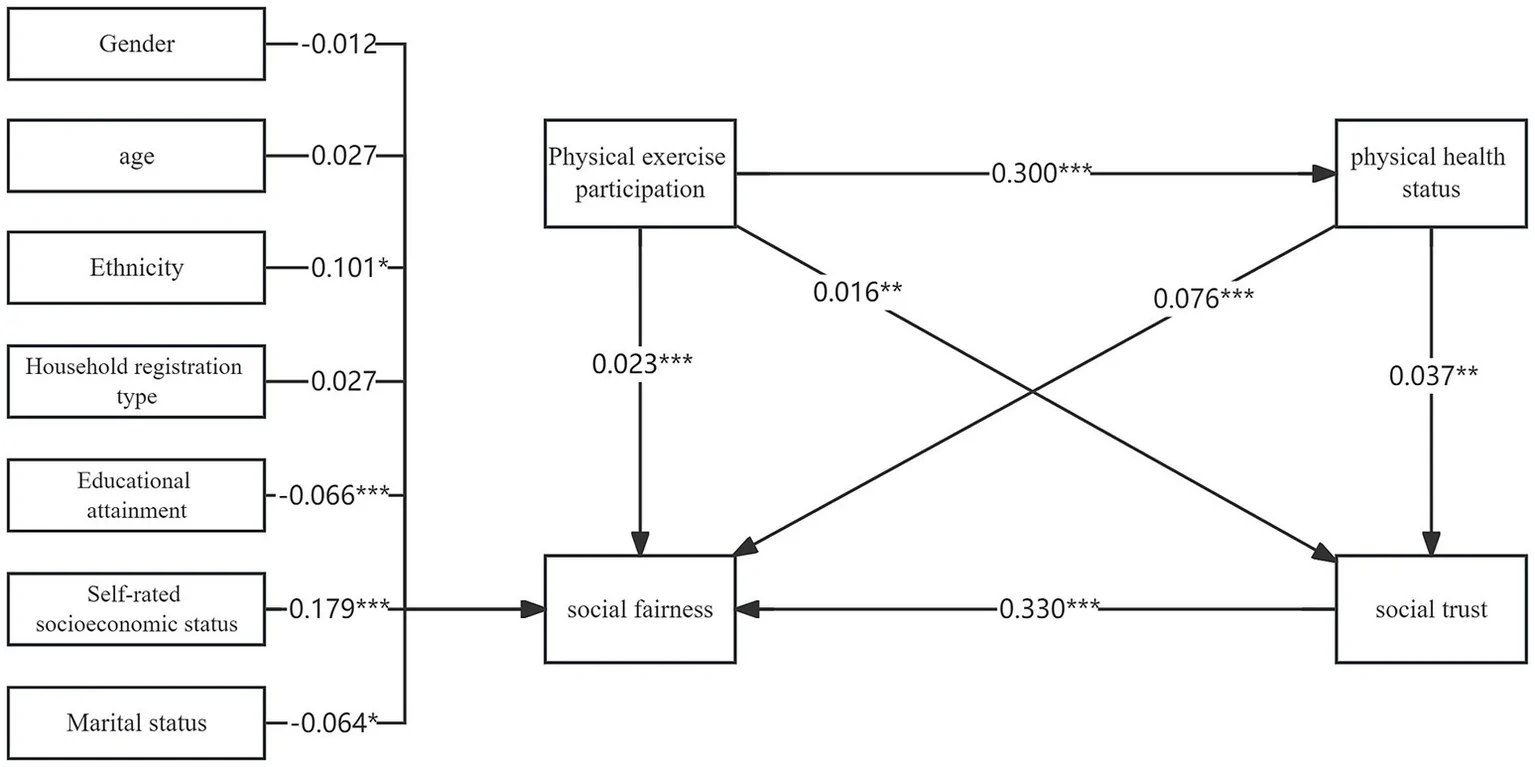
Structural equation model of the associations among physical exercise participation, self-rated physical health, social trust, and sense of social justice.

To assess whether the mediation results were sensitive to the operationalization of physical exercise participation, additional sensitivity analyses were conducted using the original ordinal exercise-frequency measure and two alternative dichotomization thresholds. As shown in Table 5, the indirect effects through self-rated physical health and social trust, as well as the serial indirect effect through self-rated physical health and social trust, remained positive and statistically significant across the alternative specifications. The total indirect effect was also statistically significant in all specifications. These findings indicate that the mediation results were robust to alternative operationalizations of physical exercise frequency and were not driven by the dichotomization threshold adopted in the main analysis.

**Table 5:** Sensitivity analyses using alternative specifications of physical exercise frequency.

Indirect effect	Main binary specification	Original ordinal specification	Monthly or more	Weekly or more
Exercise → self-rated physical health → social justice	0.0228 [0.0142, 0.0326]	0.0058 [0.0035, 0.0084]	0.0177 [0.0109, 0.0264]	0.0141 [0.0081, 0.0217]
Exercise → social trust → social justice	0.0054 [0.0012, 0.0096]	0.0026 [0.0009, 0.0043]	0.0075 [0.0028, 0.0122]	0.0077 [0.0025, 0.0129]
Exercise → self-rated physical health → social trust → social justice	0.0036 [0.0010, 0.0069]	0.0009 [0.0002, 0.0017]	0.0028 [0.0006, 0.0053]	0.0022 [0.0006, 0.0045]
Total indirect effect	0.0318 [0.0026, 0.0610]	0.0093 [0.0026, 0.0160]	0.0281 [0.0074, 0.0499]	0.0240 [0.0025, 0.0449]

Values are unstandardized indirect effects, with 95% bias-corrected Bootstrap confidence intervals in brackets. The main binary specification distinguishes any exercise participation from no participation. The original ordinal specification retains the original exercise-frequency categories, with higher scores indicating more frequent physical exercise. “Monthly or more” and “Weekly or more” represent alternative binary specifications based on increasingly stringent exercise-frequency thresholds. Indirect effects were estimated using 5,000 Bootstrap resamples.

As a sensitivity analysis, an alternative serial model was tested in which social trust preceded self-rated physical health (Table 6). The reverse serial indirect association (physical exercise participation → social trust → self-rated physical health → sense of social justice) was not statistically significant (indirect effect < 0.001, 95% CI [−0.001, 0.003]). Similarly, the specific indirect association through social trust alone was not significant in this alternative specification (indirect effect = 0.009, 95% CI [−0.011, 0.030]), whereas the indirect association through self-rated physical health remained significant (indirect effect = 0.023, 95% CI [0.014, 0.033]). Thus, the alternative ordering received limited statistical support. However, because the data are cross-sectional, these findings should not be interpreted as establishing the temporal ordering of social trust and self-rated physical health.

**Table 6:** Sensitivity analysis of the alternative serial model.

Category	Effect value	Bootstrap SE	95% CI	Relative mediating effect/%
Total indirect effect between physical exercise participation and sense of social justice	0.032	0.012	[0.010, 0.055]	58.28%
Physical exercise participation → self-rated physical health → sense of social justice	0.023	0.005	[0.014, 0.033]	41.62%
Physical exercise participation → social trust → sense of social justice	0.009	0.010	[−0.011, 0.030]	16.52%
Physical exercise participation → social trust → self-rated physical health → sense of social justice	<0.001	<0.001	[−0.001, 0.003]	0.14%
Direct effect between physical exercise participation and sense of social justice	0.023	0.030	[−0.037, 0.083]	41.72%

## 4 Discussion

This study identified a significant positive association between physical exercise participation and sense of social justice among Chinese residents. Physical exercise may be relevant to fairness perceptions not only as a health-related behavior but also as a form of social participation. Previous research has linked sport-related fairness perceptions with supportive attitudes (34), while physical exercise is also associated with social connections, social support, community engagement, and social cohesion (10, 35, 36). From a social capital perspective, exercise participation may therefore be embedded in interpersonal interaction and social integration, which are related to individuals’ broader evaluations of the social environment. However, given the cross-sectional design, this association should not be interpreted causally. Although the association between physical exercise participation and sense of social justice was statistically significant, its standardized coefficient was small (*β* = 0.011), indicating limited practical magnitude at the individual level. In contrast, social trust showed a substantially larger association with sense of social justice (*β* = 0.331). This suggests that exercise participation should be understood as a modest correlate of fairness perceptions rather than a dominant explanatory factor, whereas social trust appears to have greater substantive relevance within the present model.

Self-rated physical health showed a significant indirect association between physical exercise participation and sense of social justice. From a health capital perspective, better health is associated with greater functional capacity and fewer constraints on work and social participation (37), whereas poorer health may coincide with greater medical burdens, restricted opportunities, and lower quality of life (38). Physical health may therefore represent an important individual resource associated with both exercise participation and fairness perceptions. Nevertheless, reverse associations are also plausible, as healthier individuals may be more likely to exercise and more positive social evaluations may themselves be related to better subjective health.

Social trust also formed a significant indirect pathway. This finding is consistent with previous evidence linking social trust to sense of social justice (28). From Social Capital Theory, exercise participation may provide opportunities for communication, cooperation, and reciprocal interaction, which are associated with generalized trust. Greater trust may, in turn, correspond to more favorable evaluations of social arrangements. However, this relationship may depend on the social context of exercise, because different forms of participation provide different opportunities for interaction and trust formation (6).

The serial association involving self-rated physical health and social trust was also supported. Physical exercise is associated with better health (39, 40), while health and social trust are positively related (41, 42). These findings suggest that health capital and social capital may represent interconnected resources associated with sense of social justice. At the same time, alternative directions remain plausible: higher trust may coincide with better health, while sense of social justice may itself be associated with both trust and subjective health. Accordingly, the SEM pathways should be understood as theoretically specified contemporaneous associations rather than evidence of temporal ordering. Longitudinal or cross-lagged studies are needed to clarify these reciprocal relationships. The theoretical significance of these findings lies less in confirming the individual associations between exercise, health, and trust than in showing that health capital and social capital can be considered jointly when explaining broader social evaluations. The significant independent and serial indirect associations suggest that health-related and relational resources may represent interconnected dimensions of the social context in which exercise participation and sense of social justice are associated. Moreover, the substantially larger association of social trust with sense of social justice than that of exercise participation itself suggests that the theoretical relevance of exercise may lie less in its direct association with fairness perceptions and more in its position within a broader configuration of health and social resources. Moreover, the alternative model showed a less consistent pattern of indirect associations than the hypothesized model. However, the cross-sectional design precludes firm conclusions about the temporal ordering of social trust and self-rated physical health.

Finally, physical exercise participation differed across age, education, hukou status, and economic status (43). These differences may reflect inequalities in resources, leisure time, health awareness, and living environments (44). Expanding access to facilities, reducing participation costs, strengthening community-based organization, and providing health guidance may help reduce participation barriers among disadvantaged groups (45). However, these policy implications should be interpreted cautiously because the present findings are cross-sectional.

## 5 Conclusions and recommendations

### 5.1 Research conclusions

Based on CGSS 2023 data, this study examined the relationship between physical exercise participation and sense of social justice among Chinese residents, as well as the indirect associations involving self-rated health and social trust. The main conclusions are as follows.

First, Chinese residents’ sense of social justice was generally above the medium level, and the proportion of residents who perceived society as fair was significantly higher than that of those who perceived society as unfair. The physical exercise participation rate in the sample was 57.11%, but clear differences existed across different groups.

Second, physical exercise participation was significantly and positively associated with sense of social justice, supporting Hypothesis 1. Third, physical exercise participation was significantly associated with better physical health status, and physical health status was further positively associated with sense of social justice, supporting Hypothesis 2. Fourth, physical exercise participation was significantly and positively associated with social trust, and the independent mediating effect of social trust was supported, supporting Hypothesis 3. Fifth, physical health status and social trust played a chain-mediating role in the relationship between physical exercise participation and sense of social justice, indicating that physical exercise participation was associated with better health status and higher social trust, which were further related to stronger sense of social justice, supporting Hypothesis 4.

### 5.2 Policy recommendations

The public service system for national fitness should be further improved, with particular attention to reducing barriers to physical exercise participation. Public sports facilities and services should be distributed more equitably across communities, parks, rural areas, and schools so as to reduce the time and economic costs associated with participation. For older adults, low-income groups, rural residents, and individuals with poorer health status, more accessible exercise guidance, safety support, and community-based programs may help facilitate participation and potential health benefits.

The social interaction dimension of physical activity should also receive greater attention. Community-based and group-oriented exercise programs may provide additional opportunities for interpersonal contact, mutual support, and social participation. Such arrangements may be particularly relevant to the social-capital dimension identified in this study, although the present cross-sectional findings do not establish that exercise participation itself causes higher social trust.

Policy design should also address inequalities in physical exercise participation. The observed differences by age, educational attainment, hukou status, and economic status suggest that participation opportunities are not evenly distributed across social groups. Accordingly, disadvantaged and low-participation groups may benefit from more targeted support. In rural areas, this may include improved maintenance of public fitness facilities and stronger community sports organization; for groups with lower educational attainment or income, accessible health information and low-cost public-interest exercise programs may be useful; and for older adults, low-risk, low-threshold, and sustainable forms of physical activity should be prioritized.

More broadly, policy development may consider the observed interrelationships among physical exercise participation, self-rated physical health, social trust, and sense of social justice. Rather than treating physical exercise as a direct means of improving fairness perceptions, the findings suggest that it may be more appropriate to view exercise participation as one component within a broader set of health-related and social resources associated with residents’ evaluations of the social environment.

### 5.3 Limitations and future research

This study has several limitations. First, CGSS 2023 is a cross-sectional dataset, which limits causal inference. In addition, physical exercise participation was treated as a binary variable, and this study could not distinguish exercise frequency, intensity, type, or organizational form. Future research could further examine the quality of sports participation.

Second, sense of social justice, self-rated physical health, and generalized social trust were assessed using single-item measures available in the CGSS 2023. Although these items capture the global constructs examined in this study and are suitable for large-scale population surveys, single-item indicators provide limited coverage of multidimensional constructs and do not allow assessment of internal consistency. They may also be more susceptible to random measurement error and individual differences in item interpretation, which could attenuate or distort the observed associations. Future studies should employ validated multi-item scales to distinguish different dimensions of sense of social justice, health, and social trust and to improve construct validity and measurement reliability.

Finally, this study mainly examined the mechanisms of health and trust. Future research could further include variables such as social support, class identity, and relative deprivation to more comprehensively reveal the complex mechanisms through which physical exercise is associated with social mentality.

## Data Availability

The original contributions presented in the study are included in the article/Supplementary material, further inquiries can be directed to the corresponding author.
